# H_2_ production in underground coal gasification with pretreatment by non-focusing microwave

**DOI:** 10.3389/fchem.2025.1586267

**Published:** 2025-05-19

**Authors:** Lele Feng, Jie Dong, Haidong Li, Jiaxuan Sun

**Affiliations:** School of Safety Engineering, China University of Mining and Technology, Xuzhou, China

**Keywords:** underground coal gasification, microwave heating, non-focusing microwave, gas production performance, comparative study

## Abstract

**Introduction:**

Underground coal gasification (UCG) faces challenges in product quality control and combustion stability. While microwave heating enhances coal seam heat/mass transfer efficiency, existing studies prioritize focused microwaves, overlooking non-focusing radiation.

**Methods:**

Two microwave systems were used to treat coal in this paper. Gasification experiments were conducted on the treated coal. Meanwhile, its microscopic properties were tested by industrial analysis, mercury pressure method and Fourier Transform Infrared Spectroscopy (FTIR) method.

**Results:**

Firstly, non-focusing microwaves achieve more stable gasification despite comparable gas production durations. Secondly, focused microwaves enhance energy absorption and reduce coal moisture, while non-focusing radiation alters coal composition by increasing volatile matter and decreasing fixed carbon. Finally, non-focusing modes improve coal combustibility and reduce particulate emissions with minimal environmental impact, contrasting with focused microwaves limited ecological effects. Both methods similarly reduce coal porosity and pore volume.

**Discussion:**

This work compares gasification performance and physicochemical changes under both modalities, revealing three critical differences. These results provide critical insights for optimizing microwave-assisted UCG systems.

## 1 Introduction

China is the world’s largest producer and consumer of coal, accounting for 55.3 per cent of the country’s total energy consumption in 2023 (Yuan, 2021; Karakurt and Aydin, 2023; Wang et al., 2023). In recent years, China has actively pursued the diversification of its energy structure by developing and utilising new energy sources such as wind, hydrogen, and biomass (Feng et al., 2024). Nonetheless, the country’s unique situation—marked by an abundance of coal, limited oil resources, and a shortage of natural gas—indicates that coal will continue to play a crucial role in its energy landscape for the foreseeable future. Consequently, coal will dominate primary energy production and consumption, remaining vital to China’s development (Triguero-Ruiz et al., 2023; Zhu et al., 2023; Xu et al., 2023). Underground coal gasification (UCG) is an innovative technology that combines mining engineering, thermochemistry, and safety science to convert coal *in situ* through controlled combustion (Tata et al., 2019). This method significantly reduces occupational hazards by eliminating the need for traditional underground mining (Hamanaka et al., 2021). However, to achieve its full potential, we must overcome two key challenges: instability in gas product quality due to uneven heat and mass transfer, and the low permeability of the original coal structure, which limits combustion control (Prabu and Jayanti, 2014; Iwaszenko et al., 2018). Addressing these issues is essential for the successful commercialisation of UCG and a safer energy future. There are many methods available to optimise UCG. Some scholars have adjusted the ratio of gasification agents (e.g., oxygen, steam, CO_2_) to optimise the reaction path, which is effective in practical applications, but the cost of oxygen is high and the mixing ratio needs to be strictly controlled to avoid the risk of explosion. In addition, some scholars have optimised the gasifier design to enhance the heat and mass transfer, but it needs to be adapted to different geological conditions of coal seams and the design complexity is high. The use of pulsating gas flow in the gasification process is also a way to improve the UCG effect, which can expand the reaction surface area and increase the gas permeability, but the complexity of the equipment is high, while the pulse frequency and amplitude need to be precisely controlled. Taken together, most of the existing methods enhance the heat and mass transfer at the gas-solid interface, but have a limited effect on the coal. The microwave can modify the coal itself. Microwave heating refers to the role of microwave radiation. The polar molecules present within the coal body absorb microwave energy, which generates thermal effects, enhances molecular movement, and encourages the formation of fissures. This process modifies the coal seam through a high rate of heat, strong penetration, low thermal inertia, and selective, instantaneous heating, among other effects (Goyal et al., 2021; Zhao et al., 2020). At the same time, compared with traditional heat injection, microwave heating can release the water lock effect, with great potential to increase the penetration of the coal body (Huang et al., 2020; Li et al., 2017a).

Microwaves have the potential to improve the heat and mass transfer of coal, but current studies have used focused microwaves. In China, Zhang et al. (2018) used XPS to analyse the changes in the surface properties of coal during microwave heating and found that the moisture content in the coal decreased linearly and the drying efficiency was much higher than conventional heating. The low-field NMR method is capable of accurately predicting the moisture content and its state of existence in materials and is widely used in various industries. Lu et al. (2020) investigated the effect of microwave heating on water plugging damage in coal seams and found that the hydrophobicity of coal increased under the action of microwave radiation, so microwave heating can be used as a supplementary measure for hydraulic fracturing. Lin et al. (2021) found that the water in lignite appeared to evaporate in homogeneously under the condition of high-power microwave heating, revealing that the change of water content in lignite is actually the coupled migration of liquid water and water vapour. Li et al. (2017b) found that with the increase of microwave action time, the free water in fissures and macropores evaporated first, and the bound water in micropores evaporated later. Li et al. (2019) found that microwave radiation decomposed the aerobic functional groups and aliphatic hydrocarbons in the coal and increased the content of ether groups. Liu et al. (2020) found that microwave radiation promoted the breakage of the aliphatic side chains connected with aromatics in the coal from Jundong, making the coal structure more compact. Dong et al. (2023) found that with the increase of the microwave time, the content of Ar-OH in the coal increased first and then decreased, and the content of -C=O- was exactly the opposite. Hong et al. (2018) found that with the rise of microwave power, the pore connectivity first decreased and then increased, the total pore volume continued to increase, and the total specific surface area first increased and then decreased. In foreign countries, existing studies have reached more mature conclusions on the response characteristics of coal under microwave action. Tahmasebi et al. (2012) used infrared spectroscopy to characterise the molecular stacking structure of the coal and the groups it contained, and found that under microwave radiation with a maximum output power of 700 W, the aromatic carbon was basically unchanged, the oxygen-containing functional groups (hydroxyls and carbonyls) gradually decreased, and the aromaticity gradually increased. Pickles et al. (2014) found that below 100°C, water evaporation from coal is slow, and a large amount of bound water is converted to free water resulting in a gradual increase in the coal dielectric constant, when the temperature exceeds 100°C, with a large amount of water evaporation, the water saturation of the coal body decreases substantially and the dielectric constant begins to decrease. Abdelsayed et al. (2018) compared the effect of conventional heating with microwave heating on the coal coke structure and found that microwave heating leads to an increase in coal pyrolysis gas products, an increase in the CO/CO_2_ ratio and a decrease in tar yield. Ellison et al. (2022) studied the reaction products of four different coal types under CO_2_ atmosphere in a microwave reactor, finding that the CO and H_2_ yields under microwave gasification conditions were much higher than those under conventional gasification conditions. Focused microwave will not only affect the water and pore of coal, but also affect the functional groups, lattice structure and compound types in coal.

However, focused microwaves continuously propagate and reflect within the resonant cavity, thereby radiatively heating objects within the cavity (Shen et al., 2024) Focused microwaves require that the heated object be surrounded by the resonant cavity, and therefore the size of the heated object cannot be too large, which is obviously not applicable to underground coal seams. In contrast, non-focusing microwave is a form based on antenna emission and does not depend on the resonant cavity, and the non-focusing microwave is conducted to the antenna by a coaxial line and radiates to the surrounding open space, which can be used for the radiant heating of large objects (Ge et al., 2022). It allows the coal seam to be radiated directly via the gasification channel, rather than requiring the seam to be completely encased in a resonant cavity. Currently, non-focusing microwave has been applied in the heating of underground oilfield sands in the United States, Brazil, and other underground oilfields, but its effect on the modification of coal seams is lacking in research, and needs further in-depth exploration. Non-focusing microwave and focused microwave are not only the difference of electromagnetic wave propagation direction, but also the difference of electromagnetic wave reflection, superposition, resonance and other processes, as well as the resulting coal body response, dielectric loss, local heat generation and other behaviours, resulting in the analysis of water out of the coal, the expansion of the fissure, the transformation of the functional group and other micro-processes are different from the focusing microwave modification, which contains a series of unresolved scientific issues.

To this end, the paper is intended to compare the gasification reaction characteristics and physicochemical structure response law of coal under different microwave radiation conditions based on physical experiments, to elucidate the difference between the effects of focused microwave radiation and non-focusing microwave radiation on coal, and to further reveal the mechanism of non-focusing microwave action. The results of the study can provide an important basis for the enhancement of the coal’s heat and mass transfer capacity and the improvement of the quality of the gasification products in the subsurface.

## 2 Methodology

### 2.1 Preparation of coal sample

In this paper, bituminous coal from Shenmu coal mine in Shaanxi, China, was used, and the basic parameters of the coal samples are shown in Table 1. The original coal samples were cut and polished along the laminations to form a circular coal column with a diameter of 50 mm and a height of 100 mm. To ensure the accuracy of the subsequent ignition and gasification process, holes were drilled on the upper surface of the tested cylindrical coal samples, with a hole diameter of 10 mm and a hole depth of 100 mm as the initial gasification channel.

**TABLE 1:** Property of coal sample used in experiments.

Proximate analysis, %, air dry base			
M_ad_	V_ad_	A_ad_	FC_ad_
10.36	3.90	28.02	57.72

### 2.2 Microwave modification system

**FIGURE 1:**
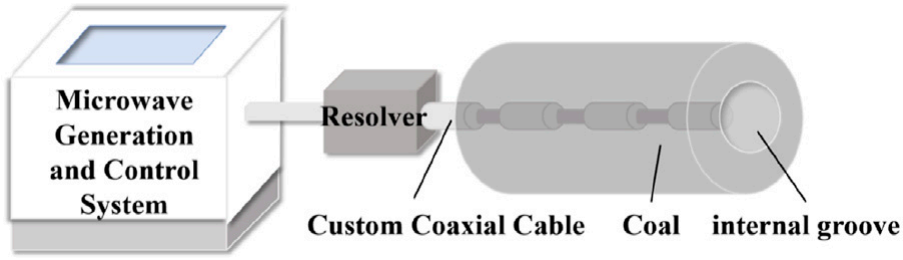
Non-focusing microwave radiation experiment system (AL-MDR01).

The non-focusing microwave modified coal body experiment uses a non-focusing microwave radiation experiment system, as shown in Figure 1.

The main feature of this AL-MDR01 Controllable Electromagnetic Wave Generation System is that it adopts a unique microwave antenna structure to emit microwaves for radiation reaction, and it adopts a 7-inch control panel for precise temperature control, easy and convenient operation, and real-time storage of data and curves. After the microwave is transmitted from the waveguide, it passes through the waveguide-coaxial converter and is changed to be propagated by the customised coaxial line. The standard coaxial line has four layers, from the inside to the outside, which are the transmission layer, the insulating layer, the shielding layer, the protection layer, and the customised coaxial line used here, which is grooved for the protection and shielding layers at intervals, so that the electromagnetic waves at the grooved place can be transmitted to be radiated to the surroundings to form a non-focusing microwave field. Customised coaxial cables are available in total lengths of 100 mm.

The device mainly consists of three main parts: microwave generating unit, wave guide-coaxial converter and customised coaxial line (microwave antenna), where the microwave generating unit in turn contains transformers, capacitors, magnetrons, water-cooling system, etc., which generates microwaves with a power ranging from 0 W to 2,000 W at a frequency of 2.45 GHz.

**FIGURE 2:**
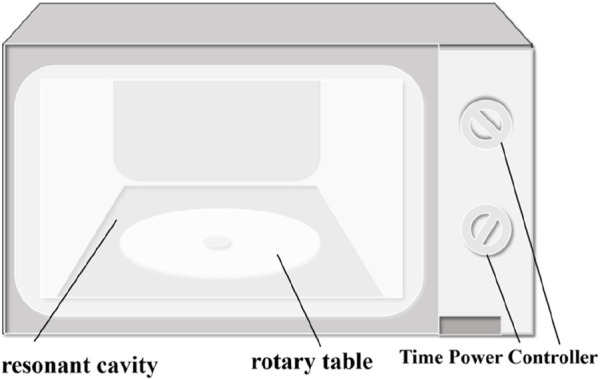
Microwave oven (Galanz, P70D20N1 P-G5).

A microwave oven (Galanz, P70D20N1 P-G5) was used for the focused microwave-modified coal body experiments, as shown in Figure 2.

The device comprises seven main parts: magnetron, resonant cavity, waveguide, rotating table, oven door, time-power controller. It can generate microwaves with a power output ranging from 300 to 700 W at a frequency of 2.45 GHz.

### 2.3 Ignition experiment system

**FIGURE 3:**
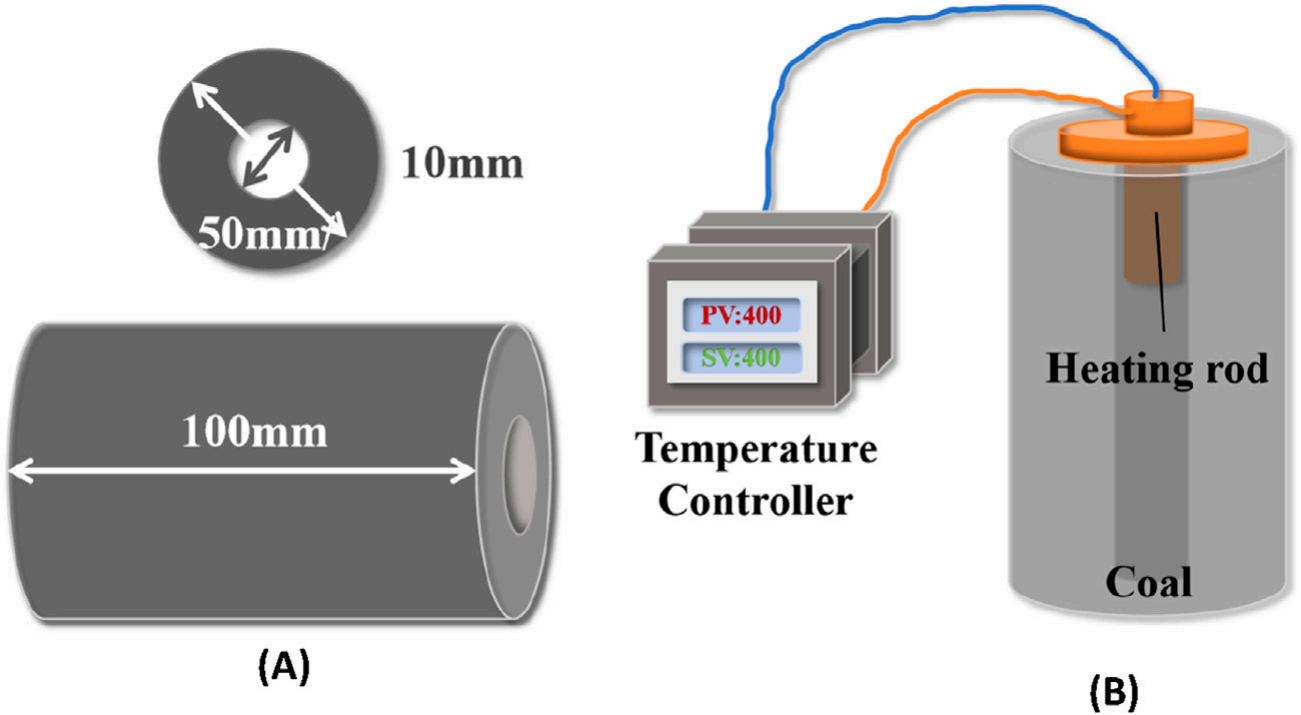
Electric heating system. **(A)** Experimental coal pillar; **(B)** Electric heating device.

The coal body ignition experiment was conducted by electric ignition, using an electric heating device to increase the temperature of the coal column, the structure of which is shown in Figure 3. The heating rod has a voltage of 220 V, a power of 200 W, a diameter of 9.5 mm, and an operating temperature of 400°C. It is connected to the temperature controller by a wire and adapted to the gasification channel of the coal column, so that it can be heated stably. According to the pre-experimental results, the ignition of the coal column takes 3 h when the heating temperature is 400°C. Therefore, in this study, the ignition parameters for all conditions were 400°C and 3 h.

### 2.4 Gasification experiment system

**FIGURE 4:**
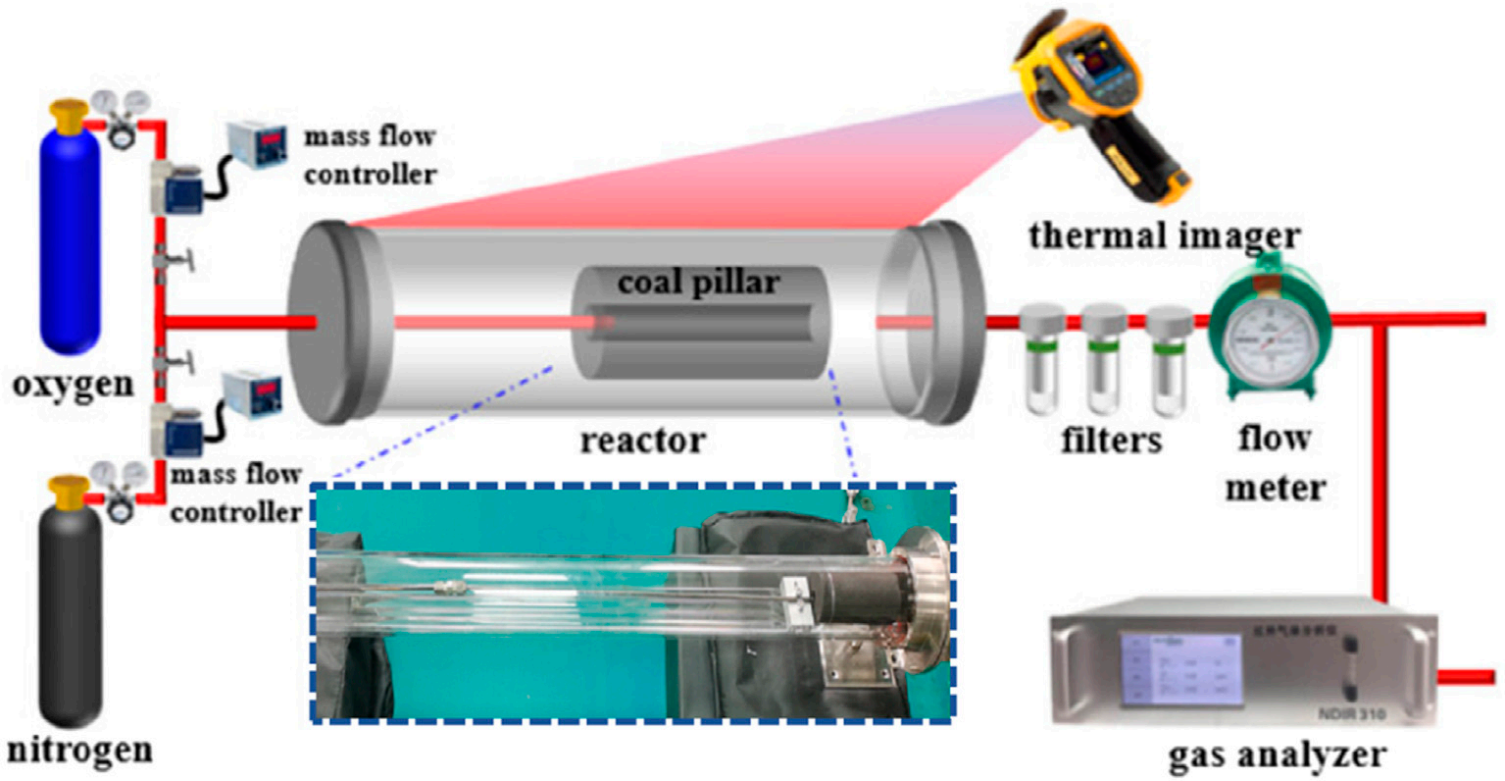
Gasification experiment system (Dong et al., 2024)

After the ignition was completed, the successfully ignited coal column was immediately fed into the reactor and injected with oxygen at a flow rate of 1 L/min and nitrogen at a flow rate of 0.8 L/min as the gasification agent to start the gasification test. The experimental system is based on a vacuum tubular furnace, which can effectively ensure the gas tightness of the gasification process, as shown in Figure 4(Dong et al., 2024) The experimental system consists of a gas supply unit, a tubular reactor, a temperature measurement unit and a gas production measurement unit. Two mass flow controllers (D077B, Sevenstar) and a matching flow display device (D08-1F, Sevensbar) were used to control the flow rates of oxygen and nitrogen, respectively, with a controller flow uncertainty of±0.15 L/min. High-temperature-resistant, highly transparent quartz tubes were used as the gasification reactor, with an inner diameter of 72 mm, an outer diameter of 80 mm, and a length of 1,000 mm. A thermal imager (Fluke Ti480 PRO) was used to convert the invisible infrared energy into a visible thermal image, and the temperature distribution of the coal column was measured every 15 minutes with an uncertainty of ±20°C. The infrared thermal imager receives infrared radiation in the 8–14 μm band emitted from the surface of the target object through a high-sensitivity VO_x_ focal plane detector, converts the radiant energy into electrical signals, and then calculates the temperature distribution by 18-bit ADC analogue-to-digital conversion and digital signal processing, combined with Stefan-Boltzmann’s law, and adopts the MSX multispectral imaging technology to integrate the visible image with the thermal map. The visible light image is fused with the thermogram using MSX multispectral imaging technology, and a pseudo-colour thermogram with temperature measurement data is finally generated. A wet flow meter (LMF-2) was used to monitor the flow rate of the gas produced over a period of time with the help of the internal mechanical moving parts, and the accuracy of the flow rate of the gas produced over a period of time was ±0.15 Lmin. The flow rate of the produced gas can be monitored with an accuracy of ±1% of full scale with the help of internal mechanical moving parts. In addition, the composition of the produced gases (O_2_, H_2_, CO, CO_2_, CH_4_, and C_n_H_m_) is measured utilising a gas analyser (YF-V01), which acquires a set of data at intervals of a few seconds and saves them. The core principle of a gas analyser is to detect and measure specific components of a gas using its internal sensor. When a target gas passes through the sensor, it causes a change in the chemical properties of the sensor, which in turn generates an electrical signal. This signal is amplified and processed to provide information about the composition and concentration of the gas. The accuracy of the measurements of O_2_, H_2_, and CO, CO_2_, and CH_4_ is ±2%, ±0.4%, and ±2%, respectively.

### 2.5 Experimental design

To reveal the influence of various microwave radiation methods on gas production and the physicochemical properties of coal, the microwave radiation power was fixed at 700W, while the microwave radiation method and duration were adjusted to examine the changes in gas production performance and microcosmic properties of coal under different conditions. The impact of microwave radiation methods on the characteristics of the gasification reaction of coal and the physicochemical structure was analysed under varying radiation times. In terms of microwave power, prior studies revealed that excessively low power results in slow coal heating, failing to reach effective modification temperatures, while excessively high power induces coal pyrolysis, compromising structural integrity. Thus, a balanced power of 700 W was selected. For irradiation time, 1 min serves as a short-duration benchmark to observe initial coal responses, 3 min represents an intermediate duration to assess thermal expansion and structural changes, and 5 min evaluates risks associated with prolonged exposure (e.g., overheating). The 2-min gradient between intervals ensures effective capture of nonlinear modification dynamics. The experimental working conditions are shown in Table 2.

**TABLE 2:** Working condition design.

No.	Microwave radiation patterns	Microwave time (min)
1	None	0
2	non-focusing	1
3	non-focusing	3
4	non-focusing	5
5	focused	1
6	focused	3
7	focused	5

## 3 Experimental results and discussion

### 3.1 Influence of different microwave radiation methods on the effect of coal gasification

**FIGURE 5:**
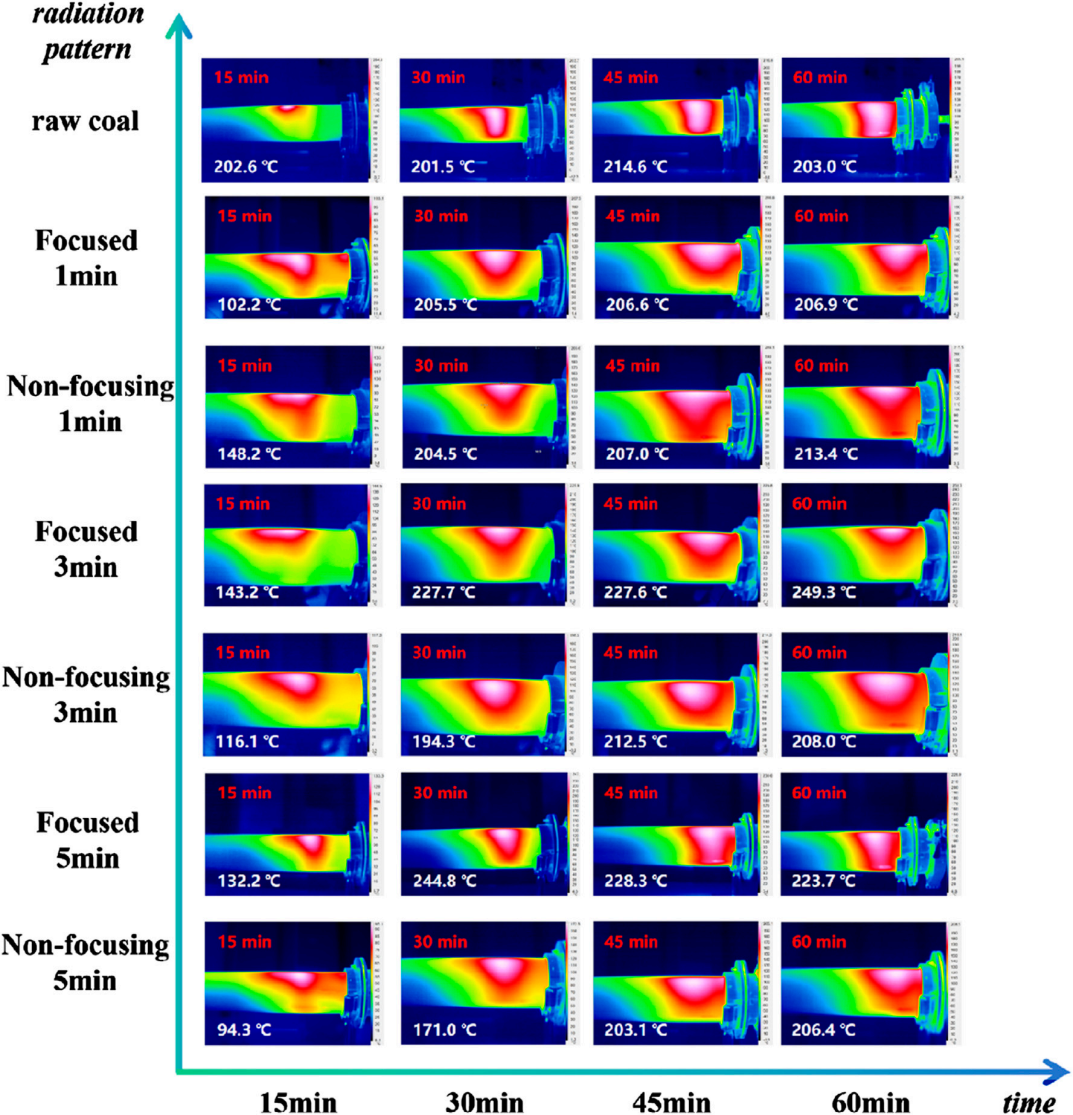
Temperature distribution under different microwave radiation conditions.

During the gasification process, the temperature of the coal column under different microwave radiation conditions was measured. The ignited coal column was put into the tube furnace, the gasification time was set to 1h, and the temperature field was characterised with the help of infrared thermography every 15 min, as shown in Figure 5, to show the warming characteristics and heat distribution in the gasification process of different coal columns after microwave radiation through the temperature distribution on the surface of the coal body, and the highest temperature was shown through the unified temperature legend.

As can be seen from Figure 5, the combustion area of the coal column in the gasification process is “pocket” type. With the extension of the combustion time, the reaction area expands along the periphery. The microwave radiation modification does not have a great influence on the maximum temperature of the coal samples at each stage of the gasification reaction period. After modifying the coal column with microwave radiation, the heating rate during the gasification process increases significantly, resulting in reduced heat loss. This improvement enhances the efficiency of gasification production. When microwave radiation lasts for 1 min or 3 min, the focused microwave has a more pronounced effect on the heating rate of the coal column, allowing for faster and more stable heating. However, at 5 min of microwave radiation, the situation changes; longer radiation times lead to the non-focusing microwave exhibiting a more stable effect on the gasification process of the coal column.

**FIGURE 6:**
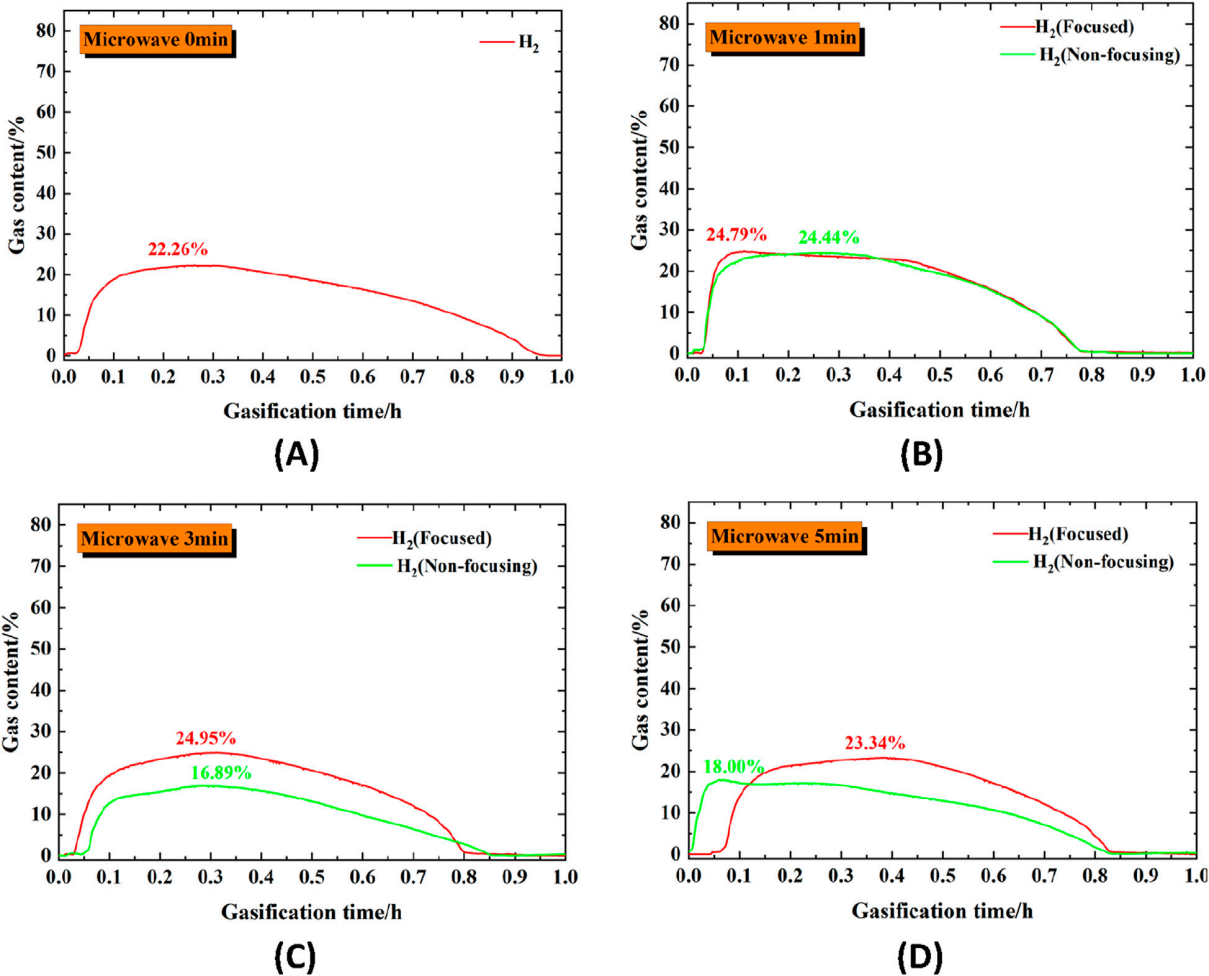
Gas composition of coal column gasification under different microwave radiation conditions. **(A)** Raw coal; **(B)** Coal after 1 min of two microwave radiation; **(C)** Coal after 3 min of two microwave radiation; **(D)** Coal after 5 min of two microwave radiation.

In order to study the effect of different microwave radiation methods on the gasification performance of coal columns, a gas analyser was used to record the gases produced during the gasification of coal columns. H_2_, as an important gas product produced during the process, was studied separately. Figure 6 visualises the H_2_ gas production during the coal gasification process under different conditions.

The overall gasification process was analysed. Under all conditions, the H_2_ content reached a rapid maximum after the start of the reaction, followed by a steady output for a period of time and then continued to decay to zero. Corresponding to the aforementioned heating rate of the coal column during the gasification process, the reaction rate of the coal increased, and the effective gas production time decreased after microwave radiation treatment. The effective gas production time of the original coal reaches 0.9h; when the microwave radiation time is 1min, the effective gas production time of the coal column under the two microwave radiation methods is 0.75h; when the microwave radiation time is 3min, the effective gas production time of the coal column under the two types of microwave radiation methods is 0.8h; when the microwave radiation time is 5min, the effective gas production time of the coal column under the focused microwave radiation method is 0.8h, and the effective gas production time of the non-focusing microwave radiation method is 0.85h. From the microscopic results described below, it can be seen that both microwave radiation methods increase the volatile content of coal and decrease the fixed carbon content. In the gasification process, volatile matter is rapidly released when heated, forming a large number of reactive radicals and combustible gaseous intermediates, which significantly reduces the reaction activation energy and thus accelerates the gasification rate. At the same time, microwave radiation increases the content of aromatic hydrocarbons and oxygen-containing functional groups in coal functional groups. These structures are thermally unstable and can be broken at lower temperatures to produce small molecule gases (such as H_2_, CO). Therefore, the reactivity of coal increases after microwave radiation, and the gasification speed is accelerated, but the effective time may be shortened due to rapid consumption.

**FIGURE 7:**
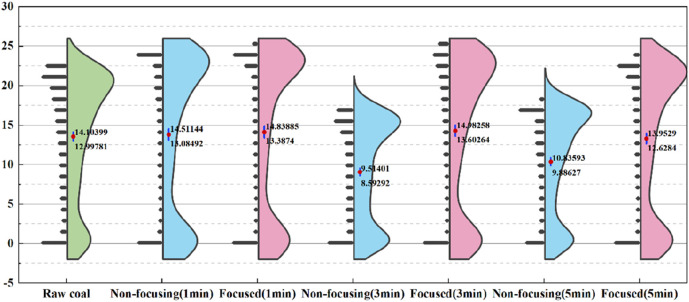
H_2_ production under different microwave radiation conditions.

To further investigate the gasification products and effects of coal columns under different microwave radiation conditions, the output characteristic curves of different gases were extracted on the basis of gasification curves. Their yields were quantitatively expressed by using 95% confidence intervals, and Figure 7 shows the visualisation results of the H_2_ yield.

As evident from the experimental data, microwave irradiation induces a marginal reduction in H_2_ yield during coal gasification. Notably, non-focused microwave treatment demonstrates optimal H_2_ yield enhancement at 1-min irradiation, whereas focused microwave irradiation achieves superior performance at 3-min exposure. Comparative analysis reveals that prolonged irradiation duration results in a more pronounced decline in H_2_ yield for non-focused microwave treatment relative to its focused counterpart.

### 3.2 Effect of different microwave radiation modes on industrial fractions in coal

Focused microwaves penetrate from the outside to the inside of the coal column, while non-focusing microwaves do the opposite. Therefore, in this study, samples were taken from the inside and outside of the coal column for microscopic tests, in order to reveal the effects of the different microwave radiation methods on the coal in a further way.

**FIGURE 8:**
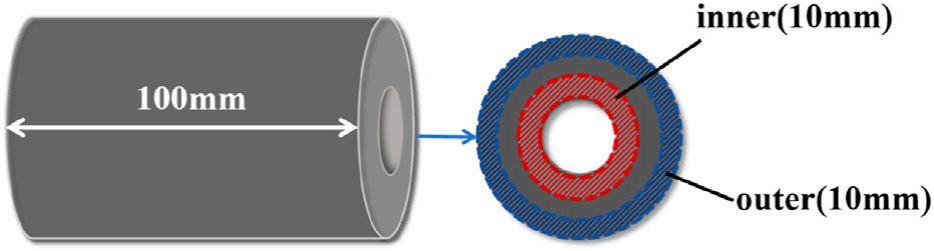
Schematic diagram of sampling areas for industrial analyses of coal.

**FIGURE 9:**
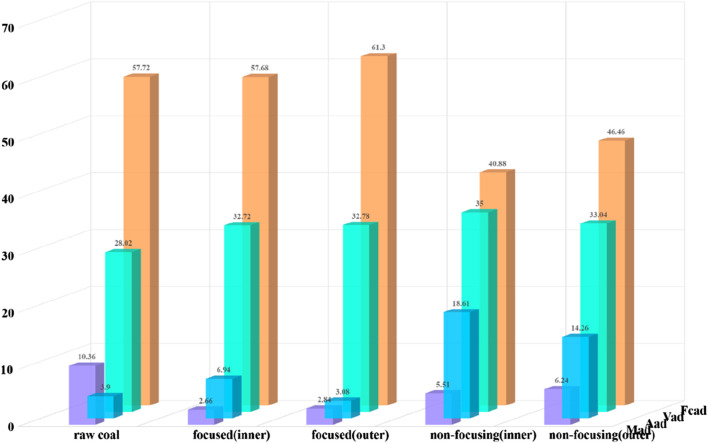
Results of industrial analyses of coal samples.

The effect of microwave radiation on the industrial components of coal was investigated by determining the moisture, volatile matter, ash and fixed carbon composition of coal through industrial analysis. To investigate the penetration effect of different microwave methods on the coal column, samples were taken from the inside and outside of the coal column respectively, and the area is shown in Figure 8. A certain weight of coal samples was weighed and dried in a drying oven at 45°C–50°C for 8 h, then removed and cooled. The weight of moisture lost after drying as a percentage of the original weight of the coal samples is called the external moisture. Loss of external moisture in the above coal samples at 102°C–105°C continues to dry for 2 h. The weight loss percentage of the specimen, calculated relative to its initial mass, represents the internal moisture content. The sample is sealed in a crucible and placed in a muffle furnace at 850°C for 7 min to allow the volatiles to escape, removed and placed in a desiccator to cool to room temperature and then weighed. The percentage of the weight lost that accounts for the original weight of the sample is called the volatile fraction. The remaining part of the coal sample after the loss of water and volatile matter is coke. It consists of fixed carbon and ash. The coke is burnt below 800°C ± 20°C until constant weight, then removed and cooled. The weight lost by the coal is the fixed carbon, and the remaining part is the ash. The weight percentage of these two parts to the original specimen is the content of fixed carbon and ash, and the results of the industrial analysis of the coal samples are shown in Figure 9.

As can be seen from Figure 9, the moisture in the coal decreases after the microwave radiation effect, the ash and volatile content increase, and the fixed carbon content changes with the change of microwave radiation mode. When microwave radiation is applied, the higher water content in coal can absorb a large amount of microwave energy, so that the internal temperature of the coal body, water evaporation, and have an impact on the moisture content in the coal body are affected. Compared with non-focusing microwave, the water content in the coal column after focused microwave radiation is lower. When the microwave radiation time is the same, the focused microwave can release more energy, which is conducive to the storage, transport and combustion of coal, and it can reduce the transport cost and improve the combustion efficiency; the non-focusing microwave radiation can greatly increase the ash content of the coal, and the effect of the focused microwave radiation is not very obvious, and it lowers the coal quality to a certain degree; non-focusing microwave radiation can greatly increase the ash content of the coal, and the effect of focused microwave radiation is not very obvious, which reduces the coal quality; non-focusing microwave radiation has a greater impact on the volatile fraction content of coal, i.e., coal is easier to ignite; non-focusing microwave radiation can significantly reduce the fixed carbon content of coal, even if the coal in the combustion process produces nitrogen oxides (NO_x_) and other pollutants to facilitate the reduction of environmental pollution, while the role of focused microwave is not significant.

### 3.3 Effects of different microwave radiation modes on the molecular structure of coal

**FIGURE 10:**
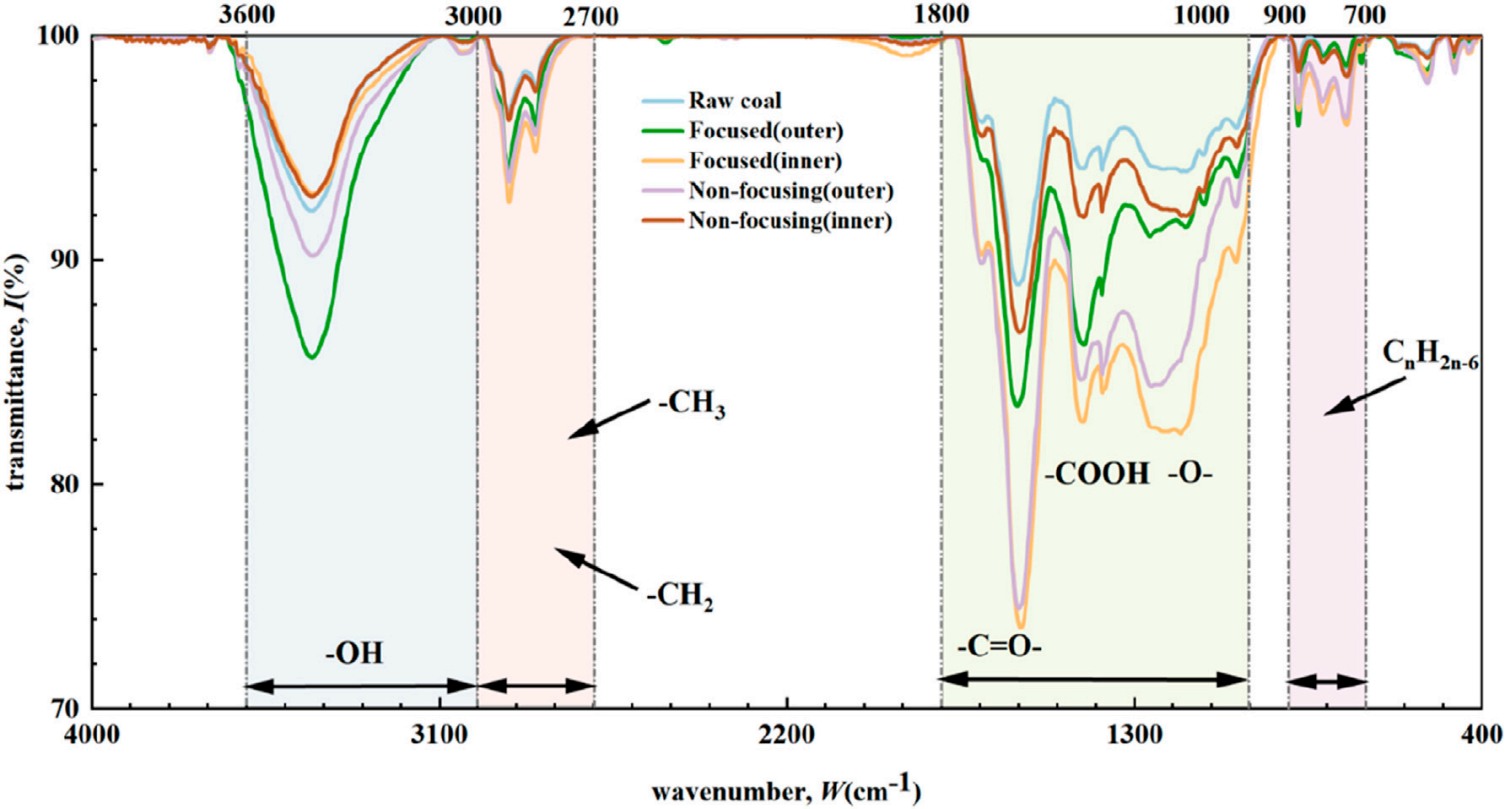
Fourier transform infrared spectra of coal samples under different microwave radiation conditions.

The Fourier Transform Infrared Spectroscopy (FTIR) method is widely used in the study of the molecular structure of coal bodies due to its fast operation and low cost. In this paper, this method is used to measure the types and contents of functional groups in coal under different microwave radiation conditions. The infrared spectrum analysis of coal samples can be divided into four parts: aromatic hydrocarbon structure region (700-900cm^-1^), oxygen-containing functional group region (1,000-1800cm^-1^), aliphatic hydrocarbon structure region (2700-3000cm^-1^) and hydroxyl structure region (3000-3600cm^-1^) (Xie et al., 2019). Figure 10 shows the Fourier transform infrared (FTIR) spectra of coal samples under different microwave radiation conditions. As can be seen from Figure 10, after microwave radiation, the -O-H content increased slightly, the -C=O- content increased, and the aliphatic content increased slightly. For -O-H, the external content of the coal column increased and the internal content remained basically unchanged after both types of microwave radiation; for -C=O-, the internal content of the coal column by focused microwave radiation and the external content of the coal column by non-focusing microwave radiation increased substantially, with an overall upward trend; and for the aliphatic group, the contents of both types of microwave radiation increased slightly. Microwave irradiation induces the cleavage of self-associated hydrogen bonds in hydroxyl groups, while the more reactive alcohol hydroxyl, phenolic hydroxyl, and carboxylic acid groups undergo dehydrogenation and thermal conversion to stable ether linkages, thereby reducing the overall hydroxyl content in coal. The -C=O- functional groups primarily exist in carbonyl, carboxyl, ester, and quinone configurations, with experimental evidence indicating that carboxyl groups exhibit the highest thermal lability while quinone groups demonstrate the greatest stability under elevated temperatures. Furthermore, microwave radiation promotes the decomposition and volatilisation of aliphatic hydrocarbon components in coal. The microwave radiation causes the decomposition of aliphatic hydrocarbons in coal and their removal in the form of volatile components, and the shortening of the fatty side branch chain.

### 3.4 Effects of different microwave radiation modes on coal pore structure

**FIGURE 11:**
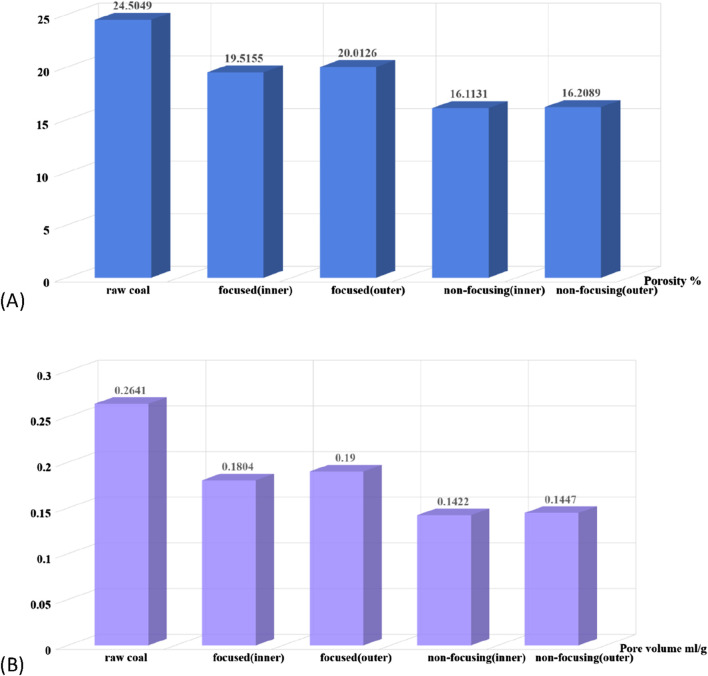
Results of mercury compression tests on raw coal and microwave-irradiated coal samples. **(A)** porosity; **(B)** pore volume.

The microwave thermal effect leads to the evaporation of free water in the pore cracks of the coal body, the thermal removal of bound water in the coal matrix. Under the influence of the difference between internal and external water vapour pressure, the structure of coal pores changes. The mercuric pressure method is a classical method widely used to characterise the pore structure of coal rocks, which can effectively obtain key parameters such as the pore size distribution, total pore volume and total pore specific surface area of the coal body. The method is based on the non-wetting property of mercury to solid materials, and the mercury is forced into the pore space of the material by applying external pressure. As the mercury pressure increases, the pore size that mercury can enter becomes smaller. The pore volume distribution in the corresponding pore size range can be determined by accurately measuring the amount of mercury under different pressure conditions. In this study, the porosity and pore volume of coal pillars under different microwave radiation conditions were determined by the mercury pressure method (Toda and Toyoda, 1972). The results are shown in Figure 11.

As can be seen from Figure 11, the pore structure of the coal body changed significantly after microwave radiation, and the average pore diameter, porosity and pore volume showed an overall decreasing trend. Compared with the focused microwave radiation, the porosity under the action of non-focusing microwave radiation was reduced from 24.50% to 16.11%, with a larger magnitude; the pore volume after non-focusing microwave radiation was reduced from 0.26 to 0.14, and that of focused microwave radiation was reduced to 0.18, with a similar effect. During the expansion of the pores of the coal body, there is an energy imbalance between the released elastic strain energy and the absorbed part of the microwave energy and the surface energy required for the newly generated surfaces, which drives the continued expansion of the pores. A part of the energy absorbed by the coal body can lead to part of the original closed pores in the coal body obtaining energy and opening, so that the porosity of the coal body will be enlarged. However, after a longer period of microwave radiation, the coal skeleton inside the coal samples contracted, which led to the closure of some pores in the coal samples and the decrease of the porosity, and the changes of the pore diameter and the pore volume and the porosity had a high degree of synchronicity.

### 3.5 Repeatability verification

**FIGURE 12:**
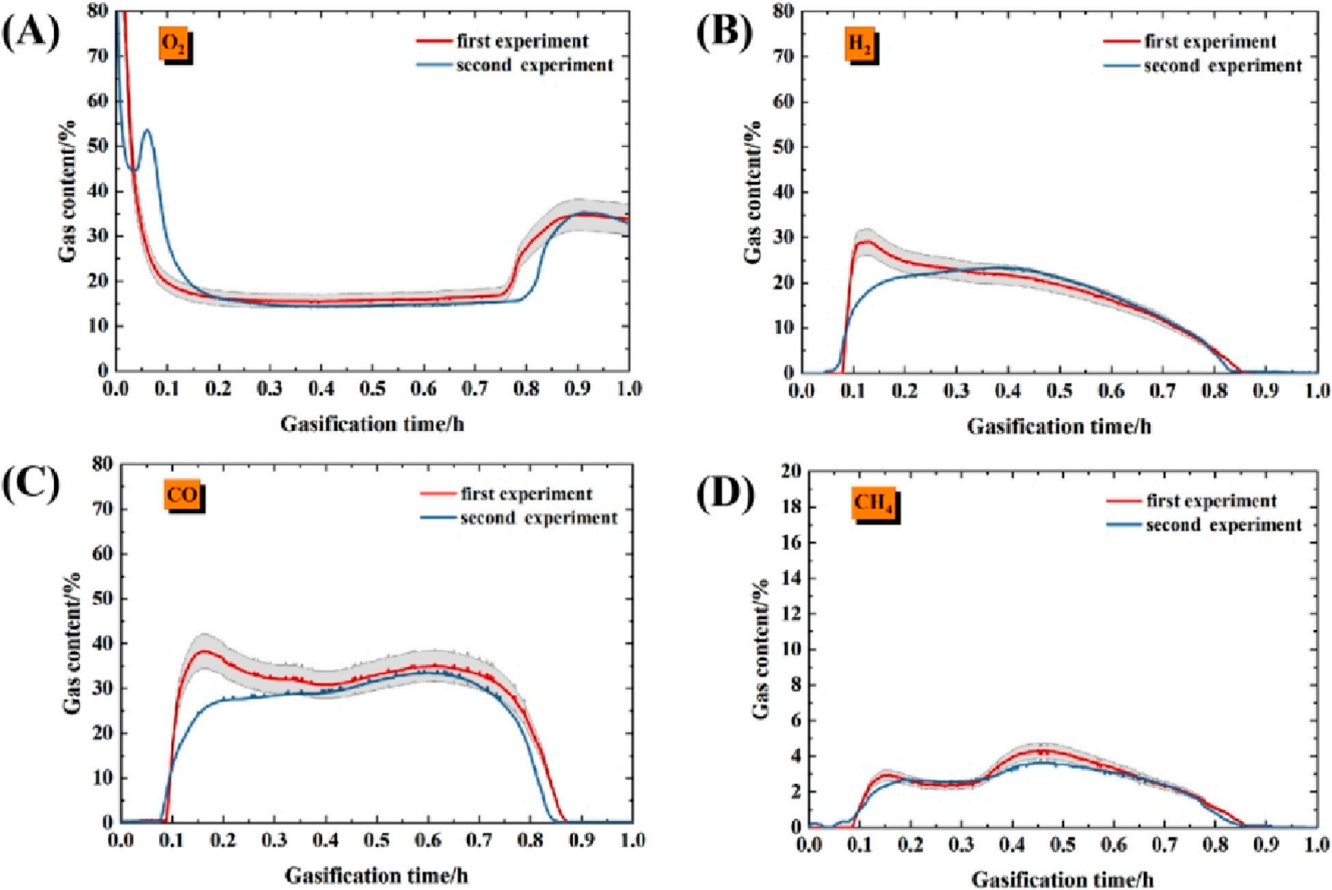
Gas production results under repetitive experiments. **(A)** Oxygen; **(B)** Hydrogen; **(C)** Carbon monoxide; **(D)** Methane.

Before experiments, repetitive experiments on the same operating conditions (300°Cfor 3 h in ignition process, straight flow and 1 L/min O_2)_ were conducted. It is found that the gas results in repetitive experiments also have strong similarity, all within the measurement error range of the instrument, as shown in Figure 12. Therefore, based on gas data, it is demonstrated that the experimental results have strong repeatability.

### 3.6 Discussion on the limitations

(1) In this paper, only one power is fixed and the microwave radiation time is changed to carry out experiments under fewer working conditions. There is a lack of analysis of the effect of different factors on the characteristics of microwave-modified coal gasification. In the future research, the microwave time and microwave power will be used as independent variables to study their effects on the hydrogen production characteristics of coal and optimise the best parameters.
(2) This paper basically focuses on the parameter changes during the gasification process and lacks the research and analysis of the microwave stage and ignition stage. In further studies, attention will be paid to the temperature rise of the coal column before and after microwave in order to reveal the difference in thermal effects of different microwave methods.
(3) In this paper, the non-homogeneity of the physicochemical properties may lack consideration when using coal pillars as the research object. Therefore, it is necessary to take coal powder as the research object in the subsequent study to analyse the gasification characteristics and compare the measurements of physicochemical properties.

## 4 Conclusion

In this study, various types of physical tests were used to compare the gasification reaction characteristics and physicochemical structure response law of coal under different microwave radiation conditions, elucidating the difference between the effects of focused microwave radiation and non-focusing microwave radiation on coal. The main conclusions are as follows.

(1) Focused microwave irradiation demonstrated superior heating efficiency during initial gasification stages (1–3 min), enabling faster temperature rise and more stable heating processes. Prolonged irradiation (≥5 min) reversed this trend, with non-focusing microwave showing greater stability in maintaining gasification reactivity.
(2) Both irradiation modes exerted comparable effects on effective syngas generation duration. However, non-focused microwave induced more pronounced H_2_ reduction in product gas compared to focused irradiation.
(3) Focused irradiation significantly reduced moisture content while releasing higher energy density per unit time. Non-focusing irradiation enhanced volatile matter content and decreased fixed carbon, improving coal ignitability and combustion stability with reduced environmental impact. Focused irradiation showed minimal effects on these parameters.
(4) Microwave irradiation increased -OH, -C=O-, and aliphatic group concentrations. Spatial distribution analysis revealed: OH elevation primarily occurred on coal surfaces regardless of irradiation mode; -C=O- content increased internally under focused irradiation but externally under non-focused irradiation, with overall upward trends. Aliphatic groups exhibited moderate growth under both conditions.

## Data Availability

The original contributions presented in the study are included in the article/supplementary material, further inquiries can be directed to the corresponding author.

## References

[B1] Abdelsayed V. Shekhawat D. Smith M. Link D. Stiegman A. E. (2018). Microwave-assisted pyrolysis of Mississippi coal: a comparative study with conventional pyrolysis. Fuel 217, 656–667. 10.1016/j.fuel.2017.12.099

[B2] Dong M. F. Feng L. L. Qin B. T. (2023). Characteristics of coal gasification with CO_2_ after microwave irradiation based on TGA, FTIR and DFT theory. Energy 267, 126619. 10.1016/j.energy.2023.126619

[B3] Dong M. F. Feng L. L. Qin B. T. Pang J. B. Han G. Xie J. H. (2024). A novel gas injection method with swirl flow in underground gasification for improving gas production and controlling pollution yields. Energy 297, 131351. 10.1016/j.energy.2024.131351

[B4] Ellison C. Abdelsayed V. Smith M. Shekhawat D. (2022). Comparative evaluation of microwave and conventional gasification of different coal types: experimental reaction studies. Fuel 321, 124055. 10.1016/j.fuel.2022.124055

[B5] Feng L. L. Gu Y. F. Pang J. B. Jiang L. L. Liu J. Zhou H. (2024). Risk identification and safety technology for hydrogen production from natural gas reforming. CBEN 11, 386–405. 10.1002/cben.202300049

[B6] Ge L. C. Liu X. Y. Feng H. C. Jiang H. Zhou T. H. Chu H. Q. (2022). The interaction between microwave and coal: a discussion on the state-of-the-art. Fuel 314, 123140. 10.1016/j.fuel.2022.123140

[B7] Goyal H. Sadula S. Vlachos D. G. (2021). Microwave heating of slurries. Chem. Eng. J. 417, 127892. 10.1016/j.cej.2020.127892

[B8] Hamanaka A. Su F. Q. Itakura K. Takahashi K. Kodama J. Deguchi G. (2021). Experimental study on evaluation of underground coal gasification with a horizontal hole using two different coals. Fuel 305, 121556. 10.1016/j.fuel.2021.121556

[B9] Hong Y. D. Lin B. Q. Nie W. Zhu C. J. Wang Z. Li H. (2018). Microwave irradiation on pore morphology of coal powder. Fuel 227, 434–447. 10.1016/j.fuel.2018.04.066

[B10] Huang J. X. Xu G. Liang Y. P. Hu G. Z. Chang P. (2020). Improving coal permeability using microwave heating technology—a review. Fuel 266, 117022. 10.1016/j.fuel.2020.117022

[B11] Iwaszenko S. Howaniec N. Smoliński A. (2018). Determination of random pore model parameters for underground coal gasification simulation. Energy 166, 972–978. 10.1016/j.energy.2018.10.156

[B12] Karakurt I. Aydin G. (2023). Development of regression models to forecast the CO_2_ emissions from fossil fuels in the BRICS and MINT countries. Energy 263, 125650. 10.1016/j.energy.2022.125650

[B13] Li H. Lin B. Q. Chen Z. W. Hong Y. D. Zheng C. S. (2017a). Evolution of coal petrophysical properties under microwave irradiation stimulation for different water saturation conditions. Energy fuels. 31 (9), 8852–8864. 10.1021/acs.energyfuels.7b00553

[B14] Li H. Lin B. Q. Hong Y. D. Liu T. Huang Z. B. Wang R. (2017b). Assessing the moisture migration during microwave drying of coal using low-field nuclear magnetic resonance. Dry. Technol. 36, 567–577. 10.1080/07373937.2017.1349136

[B15] Li H. Shi S. L. Lin B. Q. Lu J. X. Ye Q. Lu Y. (2019). Effects of microwave-assisted pyrolysis on the microstructure of bituminous coals. Energy 187, 115986. 10.1016/j.energy.2019.115986

[B16] Lin B. Q. Cao X. Liu T. Ni Z. Wang Z. (2021). Experimental research on water migration-damage characteristics of lignite under microwave heating. Energy fuels. 35, 1058–1069. 10.1021/acs.energyfuels.0c02416

[B17] Liu H. P. Chen T. P. Fang L. X. (2020). Evolution of char structure during non-isothermal low temperature pyrolysis of ZhunDong coal by microwave heating: a comparative study with conventional heating. J. Energy. Inst. 93, 1195–1206. 10.1016/j.joei.2019.11.003

[B18] Lu Y. Li H. Lu J. X. Shi S. L. Wang G. X. Ye Q. (2020). Clean up water blocking damage in coalbed methane reservoirs by microwave heating: laboratory studies. Process Saf. Environ. Prot. 138, 292–299. 10.1016/j.psep.2020.04.007

[B19] Pickles C. A. Gao F. Kelebek S. (2014). Microwave drying of a low-rank sub-bituminous coal. Miner. Eng. 62, 31–42. 10.1016/j.mineng.2013.10.011

[B20] Prabu V. Jayanti S. (2014). Heat-affected zone analysis of high ash coals during *ex situ* experimental simulation of underground coal gasification. Fuel 123, 167–174. 10.1016/j.fuel.2014.01.035

[B21] Shen L. Zhou J. Zhang X. Y. (2024). *In situ* investigation of the thermal characteristics of microwave resonance-induced focused hotspots in dimers for improving microwave heating uniformity. CASE Stud. Therm. Eng. 54, 104052. 10.1016/j.csite.2024.104052

[B22] Tahmasebi A. Yu J. L. Han Y. N. Yin F. K. Bhattacharya S. Stokie D. (2012). Study of chemical structure changes of Chinese lignite upon drying in superheated steam, microwave, and hot air. Energy fuels. 26 (6), 3651–3660. 10.1021/ef300559b

[B23] Tata S. Manosh C. P. Nader K. (2019)2019). Investigation of coal particle gasification processes with application leading to underground coal gasification. Fuel 237, 1186–1202. 10.1016/j.fuel.2018.10.058

[B24] Toda Y. Toyoda S. (1972). Application of mercury porosimetry to coal. Fuel 51, 199–201. 10.1016/0016-2361(72)90080-4

[B25] Triguero-Ruiz F. Avila-Cano A. Aranda F. T. (2023). Measuring the diversification of energy sources: the energy mix. Renew. Energ. 216, 119096. 10.1016/j.renene.2023.119096

[B26] Wang D. L. Tian C. C. Mao J. Q. Chen F. (2023). Forecasting coal demand in key coal consuming industries based on the data-characteristic-driven decomposition ensemble model. Energy 282, 128841. 10.1016/j.energy.2023.128841

[B27] Xie J. N. Ni G. H. Xie H. C. Li S. Sun Q. Dong K. (2019). The effect of adding surfactant to the treating acid on the chemical properties of an acid-treated coal. POWDER Technol. 336, 263–272. 10.1016/j.powtec.2019.08.039

[B28] Xu F. Y. Hou W. Xiong X. Y. Xu B. R. Wu P. Wang H. Y. (2023). The status and development strategy of coalbed methane industry in China. Explor. Dev+. 50, 765–783. 10.1016/S1876-3804(23)60427-6

[B29] Yuan L. (2021). Study on the development strategy of coal mine safety in China. China coal. 47 (06), 1–6. 10.19880/j.cnki.ccm.2021.06.001

[B30] Zhang N. N. Zhou C. C. Xia W. C. Nguyen A. V. (2018). Volatilization of mercury in coal during conventional and microwave drying and its potential guidance for environmental protection. J. Clean. Prod. 176, 1–6. 10.1016/j.jclepro.2017.12.131

[B31] Zhao Q. H. Zhao X. B. Zheng Y. L. Li J. C. He L. He J. L. (2020). Heating characteristics of igneous rock-forming minerals under microwave irradiation. Int. J. ROCK Mech. Min. 135, 104519. 10.1016/j.ijrmms.2020.104519

[B32] Zhu H. Y. Jiang M. H. Zhang D. D. Goh H. H. Wang S. Y. Mao D. J. F. (2023). Carbon neutrality pathways exploration-A state-of-the-art study: key technological advancements, current challenges, and potential opportunities. Sustain. Energy. Techn. 60, 103489. 10.1016/j.seta.2023.103489

